# Time Delta Head Impact Frequency: An Analysis on Head Impact Exposure in the Lead Up to a Concussion: Findings from the NCAA-DOD Care Consortium

**DOI:** 10.1007/s10439-022-03032-w

**Published:** 2022-08-06

**Authors:** Jack Seifert, Alok S. Shah, Jaroslaw Harezlak, Steven Rowson, Jason P. Mihalik, Larry Riggen, Stefan Duma, Alison Brooks, Kenneth L. Cameron, Christopher C. Giza, Joshua Goldman, Kevin M. Guskiewicz, Megan N. Houston, Jonathan C. Jackson, Gerald McGinty, Paul Pasquina, Steven P. Broglio, Thomas W. McAllister, Michael A. McCrea, Brian D. Stemper

**Affiliations:** 1 Joint Department of Biomedical Engineering, Marquette University and Medical College of Wisconsin, Milwaukee, WI USA; 2 Department of Neurosurgery, Medical College of Wisconsin, Milwaukee, WI USA; 3 Neuroscience Research Labs, Clement J. Zablocki Veterans Affairs Medical Center, Research 151, 5000 W. National Ave., Milwaukee, WI 53295 USA; 4 Department of Epidemiology and Biostatistics, Indiana University School of Public Health, Bloomington, IN USA; 5 Department of Biomedical Engineering and Mechanics, Virginia Tech, Blacksburg, VA USA; 6 Matthew Gfeller Center, University of North Carolina at Chapel Hill, Chapel Hill, NC USA; 7 Department of Orthopedics, School of Medicine and Public Health, University of Wisconsin, Madison, WI USA; 8 John A. Feagin Jr. Sports Medicine Fellowship, Keller Army Hospital, United States Military Academy, West Point, NY USA; 9 Departments of Neurosurgery and Pediatrics, UCLA Steve Tisch BrainSPORT Program, David Geffem School of Medicine, University of California Los Angeles, Los Angeles, CA USA; 10 Department of Sports Medicine, United States Air Force Academy, Colorado Springs, CO USA; 11 Uniformed Services University of the Health Sciences, Bethesda, MD USA; 12 Michigan Concussion Center, University of Michigan, Ann Arbor, MI USA; 13 Department of Psychiatry, Indiana School of Medicine, Indianapolis, IN USA

**Keywords:** Head impact exposure, Sport-related concussion, Concussive threshold, Traumatic brain injury, Subconcussive

## Abstract

Sport-related concussions can result from a single high magnitude impact that generates concussive symptoms, repeated subconcussive head impacts aggregating to generate concussive symptoms, or a combined effect from the two mechanisms. The array of symptoms produced by these mechanisms may be clinically interpreted as a sport-related concussion. It was hypothesized that head impact exposure resulting in concussion is influenced by severity, total number, and frequency of subconcussive head impacts. The influence of total number and magnitude of impacts was previously explored, but frequency was investigated to a lesser degree. In this analysis, head impact frequency was investigated over a new metric called ‘time delta’, the time difference from the first recorded head impact of the day until the concussive impact. Four exposure metrics were analyzed over the time delta to determine whether frequency of head impact exposure was greater for athletes on their concussion date relative to other dates of contact participation. Those metrics included head impact frequency, head impact accrual rate, risk weighted exposure (RWE), and RWE accrual rate. Athletes experienced an elevated median number of impacts, RWE, and RWE accrual rate over the time delta on their concussion date compared to non-injury sessions. This finding suggests elevated frequency of head impact exposure on the concussion date compared to other dates that may precipitate the onset of concussion.

## Introduction

The biomechanical mechanism for concussion in contact sport athletes is hypothesized to include a single high magnitude head impact, repetitive lower magnitude head impacts (i.e., head impact exposure; HIE), or a combination of the two. Understanding the mechanisms and tolerance for concussion can contribute to significant clinical and preventative advancements. Focusing on the single impact mechanism, early research in primates identified concussive thresholds that were a function of peak rotational acceleration and acceleration duration.^30, 38, 44, 45, 60^ The idea of a head acceleration threshold was translated to football to understand the biomechanics of sport-related concussion.^6, 10, 11, 13, 29, 40, 46, 49, 56, 64^ One such threshold for American football players was created by reconstructing National Football League (NFL) head impacts to determine concussive biomechanics and resulted in a concussive threshold of 70–75 g.^46^ Computational modeling of NFL head impacts resulted in a threshold of 106 g.^64^ Comparatively, analyses on collegiate head impact biomechanics measured with helmet-mounted sensors suggested a threshold was significantly greater than 82 g,^29, 40^ and a similar analysis on high school head impacts reported 96 g.^12^ Rotational acceleration magnitudes of 7900 rad/s^2^,^64^ 5582 rad/s^2^,^12^ and significantly greater than 5900 rad/s^2^^29, 40^ were also reported as potential concussive thresholds in populations of professional, high school, and college athletes, respectively.

This lack of a universal sport-related concussion threshold is due, in part, to concussive impacts demonstrating large variance in linear and rotational acceleration magnitudes between concussed individuals instrumented with on-field head impact measurement systems.^22, 28, 56^ The variance of measured concussion magnitudes may be partially attributed to measurement error.^5, 19, 23, 33, 53^ However, other factors may also play a role. One analysis on head impact accelerations in American football players found that most concussions occurred well below the initially proposed threshold, and most concussive impacts were scored as having a less than one percent risk of concussion.^56^ In the same study, uninjured athletes accumulated 4589 head impacts with acceleration magnitudes greater than the average concussive acceleration magnitude. Despite the apparent lack of correlation between incident concussion and concussive impact magnitudes, evidence of individual tolerance exists, wherein concussive impact was a significant impact for the concussed individual despite being not remarkable when compared to the magnitudes of non-concussive head impacts across all athletes. A study on 21 National Collegiate Athletic Association (NCAA) Division I football players found that for 91% of players, the concussive impact was one of the five highest acceleration magnitudes the athlete experienced during their season of injury.^50^ However, when compared to the entire population of athletes, it was found that the concussive head impacts were not different than those that did not cause a concussion. This study provided evidence that each athlete may have an individualized concussion threshold, dependent upon a number of factors that may include genetics,^35^ resilience,^43^ and biomechanical factors including concussion history^27, 31, 51^ and HIE profiles.^10, 56, 59^

The second proposed sport-related concussion mechanism, HIE, refers to the total head impact burden an athlete sustains over time. HIE may reduce individual tolerance for concussion or result in the gradual development of concussion-like symptoms. Concussed college athletes have higher exposure levels on days of concussion compared to days when they did not sustain concussion or when compared to uninjured controls.^6, 10, 48, 56^ HIE as an independent concussion mechanism is dependent on three characteristics including the total number, severity, and frequency of head impacts.^11, 15^ Prior studies demonstrated the influence of different HIE characteristics on concussion risk; several analyses finding concussed athletes had an elevated number of head impacts prior to concussion compared to their own exposure profile or to matched controls.^6, 10, 56^ Similarly, the effect of non-concussive head impact acceleration magnitudes on HIE was proposed as an injury risk function,^61^ summing concussive risks of each individual head impact over a given time period based on peak linear acceleration $\left( {\ddot{x}} \right)$ and rotational acceleration $\left( {\ddot{\theta }} \right)$.^49^ This cumulative risk function was referred to as risk weighted exposure (RWE) (Eq. 1). Concussed athletes had elevated RWE at the time of concussion compared to sessions in which a concussion did not occur or to the RWE of matched control athletes.^56, 61^ The third aspect of HIE, frequency (i.e., the number of head impacts divided by the time period in which they occur), was not investigated to the same degree as number and severity of head impacts. The metric “Head Impact Density”, a summation of the peak acceleration divided by the time from previous impact for the concussive impact and the nineteen previous head impacts, was developed to quantify HIE frequency.^10^ Head impact density in concussed athletes was elevated compared to matched controls. However, the head impact density calculation often included head impacts from the previous day which artificially reduced the influence of the first impact on the concussion date, even if it was a high magnitude impact, due to the extended time between impacts.

1 $$RWE = \sum {\frac{1}{{1 + e^{{ - \left( { - 10.2 + 0.0433*\ddot{x} + 0.000873*\ddot{\theta } - 0.000000920*\ddot{x}*\ddot{\theta }} \right)}} }}}$$

These prior studies provided evidence that HIE can influence concussion, and specifically, that frequency of subconcussive head impacts may be an important factor. Building on these findings, we compared pre-injury HIE of NCAA Division I football players on their concussion date to their own HIE from non-injury days to determine if HIE frequency, in terms of the number of head impacts sustained over a fixed time period, was elevated on concussion days. Based on the studies described above, we hypothesized that HIE frequency would be elevated for concussed athletes on their concussion date compared to non-concussion dates.

## Materials and Methods

This study was conducted to provide an analysis of the role of head impact frequency for incident concussion in National Collegiate Athletic Association (NCAA) Division I football athletes. Data included in this study were part of the Advanced Research Core (ARC) of the NCAA-DoD Grand Alliance Concussion Assessment, Research, and Education (CARE) Consortium, whose methods were previously described elsewhere.^9^ The study protocol was approved by the Institutional Review Board (IRB) at the Medical College of Wisconsin (MCW) and the US Army Human Research Protection Office (HRPO). Local sites utilized a reliance agreement with the MCW IRB. Athletes from six NCAA Division I football programs were consented and enrolled in the Head Impact Measurement (HIM) Protocol during the 2015 through 2019 fall competition seasons. Three sites participated in all five seasons; the remaining three sites contributed to the final 4 seasons.

Each enrolled athlete had their helmet (Riddell Speed or SpeedFlex) instrumented with a five degree of freedom (no rotational acceleration about the vertical axis) Head Impact Telemetry System (HIT System) (Riddell SRS, Riddell, Rosemont, IL) to monitor head accelerations during all contact practices and competitions.^23^ The system measured accelerations using six linear accelerometers inside the football helmet and computed peak component and resultant linear and rotational accelerations. Data acquisition was triggered when any single accelerometer exceeded a 9.6-g threshold. As part of normal data collection, the HIT System automatically eliminates any event with an acceleration trace not representative of helmeted head impacts as part of its false positive filtering algorithm. Those events were removed from the dataset by the algorithm before any events were made available for download by the Investigative team. Upon request from the Investigator, Riddell would review HIT System outputs for any acceleration traces that were removed from the dataset and provide those traces to the Investigator. Our team made this request any time a concussion occurred in the absence of a significant head impact. Our analysis also excluded head impacts with peak resultant linear acceleration less than 10 g. Previous studies found that non-impact dynamic movements in the athlete occur below 10 g.^25^ Head impact data, including peak resultant linear and rotational accelerations, were transmitted wirelessly to a laptop on the sidelines controlled by study team members. The data were stored in the Riddell Cloud Storage and the study team was granted access to the cloud by each individual site or by transfer from local site via a secure ftp server. Personal identifying information was removed from the data by assigning a study-specific number to each athlete. Local staff and study coordinators were responsible for assigning and maintaining the HIT System. Head impact data were collected for all contact practices and competition sessions during the fall competition seasons. This included preseason training camp, regular season contact practices, and competitions. Non-concussive head impacts were not video verified for accuracy due to limited access to practice videos and the significant number of head impacts recorded during the study.

Medical staff were provided a standard definition of concussion in line with the consensus definition from the U.S. Department of Defense (DoD) evidence-based guideline initiative, which closely parallels the American Academy of Neurology (AAN) definition.^17^ Athletes entered the CARE concussion protocol after being diagnosed with concussion. In some cases, athletes immediately reported concussion and were removed from practice or game activities. Other athletes did not report concussion until after the practice or game and continued to participate after sustaining concussion. If an athlete was not immediately removed, the time of injury was determined through post-concussion interviews conducted by local athletic medicine staff, film analysis (if available), and analysis of HIT System data. The suspected concussive impact was determined by a review of head impacts sustained around the time of concussion. No head impact data were used to make any clinical diagnoses of concussion.

### Intra-athlete Analysis of HIE Frequency

HIE frequency was analyzed for athletes that sustained concussion during study participation. Daily HIE data were analyzed separately for each athlete and included all recorded sessions for the concussed athlete during the season that they sustained a concussion. Inclusion criteria were athletes who sustained concussion, had biomechanical data that coincided with the event, the concussive event was not the first event of a session, and had head impact data for at least one other session in which concussion did not occur. Non-injury sessions with only one recorded head impact and sessions labeled as “walk-throughs” were not included in the dataset. A metric called ‘time delta’ was defined as the time elapsed from the first recorded head impact on the injury date to the time of the concussive impact. Four metrics were analyzed over the time delta: head impact frequency, head impact accrual rate, RWE, and RWE accrual rate. These time delta metrics were calculated for all non-injury sessions during each athlete’s season of injury up to and including the concussion date. To analyze HIE during non-injury sessions, an iterative windowing approach was used with bounded time. The first window started with the first head impact of a session and spanned the length of the time delta period. The time delta was then shifted to each subsequent head impact until the difference between the first head impact of that window and last head impact of the entire session was less than the time delta. The four time delta metrics were calculated for each individual window.

Head impact frequency and RWE analyses were calculated as the number of head impacts or RWE across the time delta. For the date of injury this was inclusive of the concussive head impact. Accrual rates were calculated as the number of head impacts or RWE, inclusive of the concussive impact, divided by the time delta. This association was assumed to be linear. For all sessions in which an athlete did not sustain concussion, the accrual rate was calculated as the number of head impacts or RWE an athlete experienced across the time delta window divided by the time delta. For non-injury sessions shorter than the time delta, the time difference from first to last sustained head impact was used in place of the time delta in accrual rate calculations. Day of injury metrics were compared to the time delta windows in every other non-injury contact session from the season of injury. Data are represented as the percentile of the time delta metric on the concussion date relative to the distribution of that metric for all other time delta windows on non-concussion days for that athlete during the season of injury.

### Inter-athlete Statistical Analyses

A groupwise analysis was performed to identify if significant differences (*p* < 0.05) existed at time of concussion between different contact session types in the following metrics: concussive impact linear and rotational acceleration magnitudes, time delta length, number of impacts over the time delta, percentile rank of head impacts over the time delta, head impact accrual rates (impacts/minute) over the time delta, and percentile rank of head impact accrual rates, total RWE over the time delta, RWE over the time delta percentile, RWE accrual rate (RWE/min) over the time delta, and RWE accrual rate over the time delta percentile. Session types were grouped by preseason contact practices, regular season contact practices, and competitions. The inter-athlete analysis was completed in R [R version 4.0.5 (2021–03-31)—"Shake and Throw"] using a Kruskal–Wallis test with a *post hoc* Dunn Test to compare differences in the population of concussed athletes.

## Results

During the 2015–2019 NCAA Division I football fall competition seasons, 695 athletes (1160 athlete seasons) from six different universities and service academies participated in this study. A total of 606,943 head impact events were recorded. Fifty-eight athletes sustained 61 concussions during those seasons. The concussive impact was identified for all 61 concussions.

**Figure 1:**
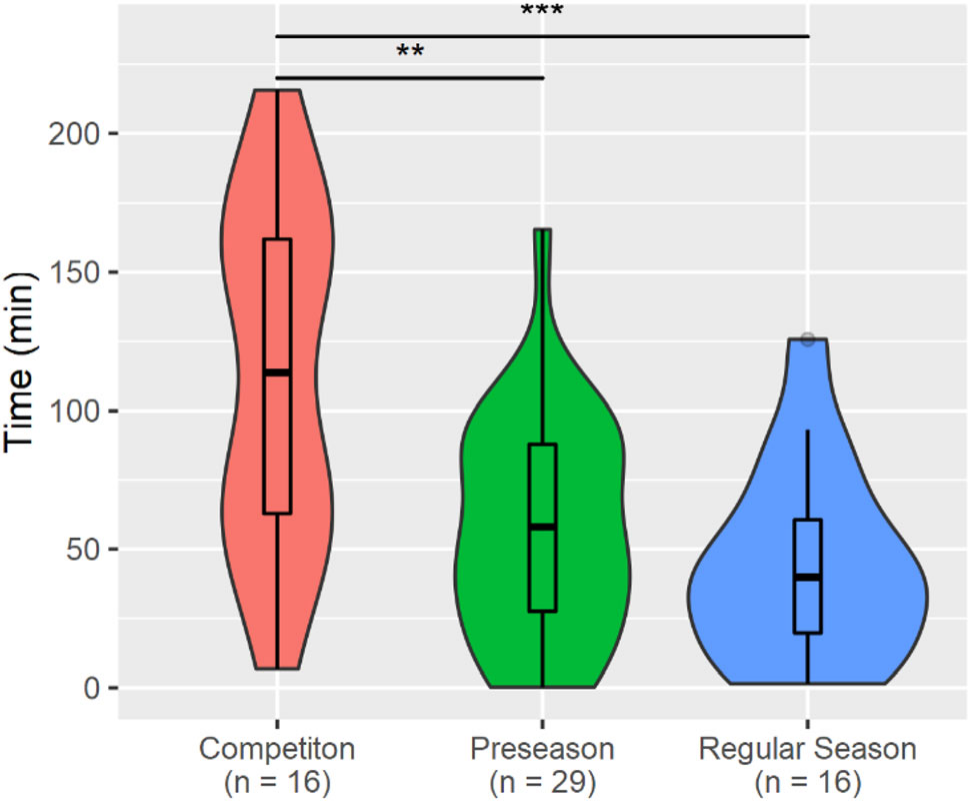
Violin plots showing each athlete's time delta (time from the first head impact of their session to the concussive event) for injuries occurring during each the three different session types: Competition, Regular Season Practice, Preseason Practice. Violin plots shown use width to show density of data points. A box plot is overlaid showing the 1st, 2nd, 3rd, and 4th quartiles. ***p-value < 0.005, **p-value < 0.01.

All concussed athletes sustained at least one head impact prior to the concussive impact on the injury date. This analysis focused on pre-concussion HIE for all 61 concussions. Accordingly, the duration of pre-concussion HIE on the injury date, as well as the number of impacts sustained and the total RWE from the first impact of that session until the concussive impact were quantified. Athlete’s time delta time lasted for significantly longer time periods in games (median = 113.65 min, interquartile range (IQR) = 99.1 min) compared to preseason practices (median = 58 min, IQR 60.4 min) (*post hoc* *p*-value = 0.006) and regular season practices (median = 39.84 min, IQR 40.9 min) (*post hoc* *p*-value = 0.0009), but no significant differences were found when comparing preseason and regular season practices (*post hoc* *p*-value = 1) (Fig. 1). During their season of injury, athletes participated in a range of 2–82 contact sessions (median = 43), inclusive of the injury date.

Linear regression analyses were performed for each contact session to justify the use of a linear assumption of head impact and RWE accumulation rates. On the day of injury, 92 and 85% of athletes had a significant linear accumulation rate (linear regression *p*-value < 0.05) for head impacts and RWE, respectively. Similar trends were found when analyzing every head impact session recorded for this study, 84 and 79% of head impact sessions had a significant linear accumulation rate (linear regression *p*-value < 0.05) for head impacts and RWE respectively.

### Time Delta Analyses

**Figure 2:**
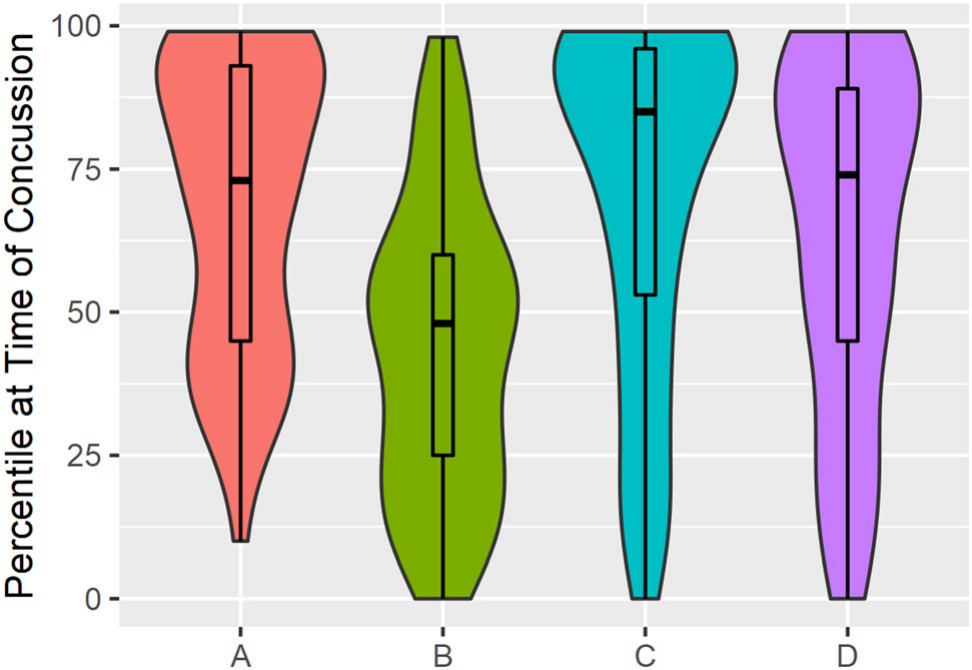
Violin plots comparing the percentile at time of injury across the population of 61 concussions for the four different time delta analyses presented, (A) Head Impact Frequency, (B) Head Impact Accrual Rate, (C) Cumulative RWE Over the Time Delta, (D) RWE Accrual Rate. Violin plots shown use width to show density of data points. A box plot is overlaid showing the 1st, 2nd, 3rd, and 4th quartiles.

**Figure 3:**
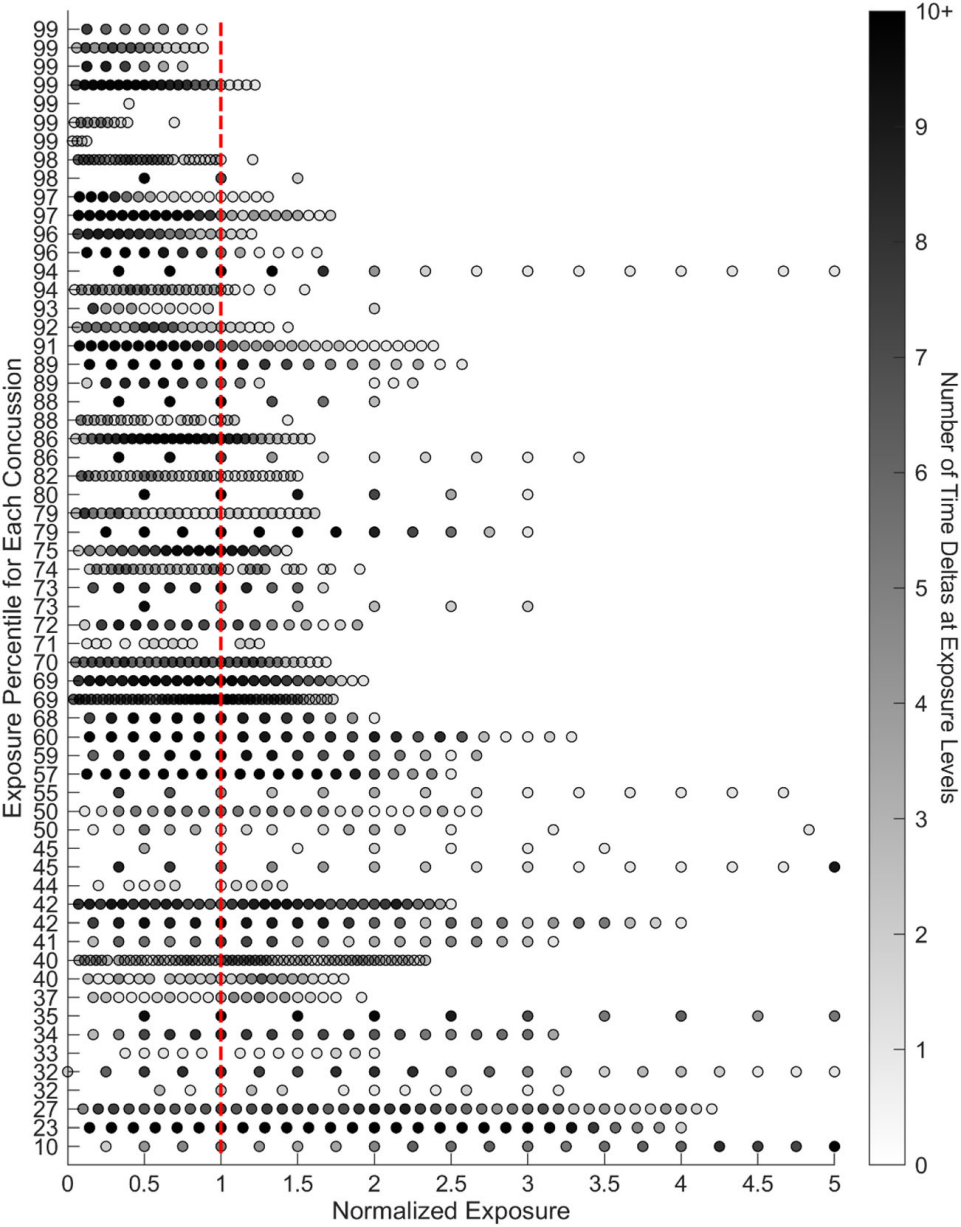
Intra-athlete analyses of the day of injury time delta impact exposure vs. all other non-injury time deltas. The number of impacts over each time delta window for every session during the season are normalized to the number of impacts over the time delta on the injury date. Each row shows the HIE history for each concussion from the season of injury. The vertical red line represents the normalized exposure on the day of injury, each dot represents a session an athlete participated in, and the shading is used to reflect the number of sessions at an exposure level. The darker the circle is equivalent to more sessions at a specific exposure level.

The four HIE metrics analyzed over each athlete’s time delta (i.e., head impact frequency, head impact accrual rate, total RWE, and RWE accrual rate) were analyzed as the percentile for that metric on the concussion date relative to the distribution of that metric across all non-injury time delta windows (median = 147 windows, IQR 234 windows) for that athlete in their season of injury (Fig. 2) (Table 1). More head impacts occurred during the time delta on the concussion date than over the time delta for any other period during that season in which a concussion did not occur (median: 73rd percentile) (Fig. 3). No difference was found in head impact accrual rates on the concussion date when compared to days that the athlete was not concussed (median: 48th percentile). Athletes also had elevated RWE (median: 85th percentile) and RWE accrual rate (median: 74th percentile) over their time delta on the concussion date compared to every other time delta period in which a concussion did not occur for that athlete during their season of injury. Because of the dependence of RWE on acceleration magnitudes, these findings suggest that the number of impacts as well as the magnitude of linear and rotational accelerations were elevated on the injury date.

**Table 1:** Results of time delta analyses showing the median percentile on the day of injury compared to all non-injury days for the athletes and number of concussions with a metric at or above the *X*th percentile.

Analysis	Median percentile	Number of Athletes above the					
		25th	50th	75th	90th	95th	99th
Number of head impacts	73	59 (96.7%)	44 (72.1%)	29 (47.5%)	18 (29.5%)	13 (21.3%)	7 (11.4%)
Head impact accrual rate	48	46 (75.4%)	27 (44.2%)	8 (13.1%)	2 (3.2%)	1 (1.6%)	0 (0%)
Total RWE	85	53 (86.9%)	46 (75.4%)	35 (57.4%)	25 (40.9%)	19 (31.1%)	11 (18%)
RWE accrual rate	74	52 (85.2%)	43 (70.5%)	28 (70.5%)	15 (24.6%)	12 (19.6%)	6 (9.8%)

### Inter-athlete Analyses

**Figure 4:**
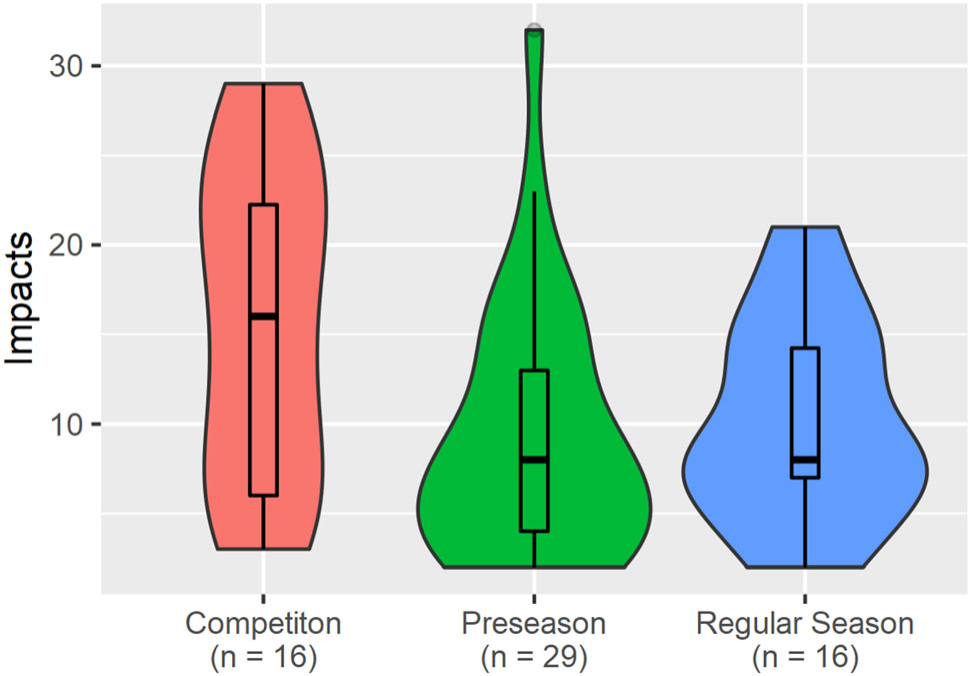
Violin plots showing the number of head impacts at the time of concussion (inclusive of concussive impact) for injuries occurring during each of the three session types: Competition, Regular Season Practice, Preseason Practice. Violin plots shown use width to show density of data points. A box plot is overlaid showing the 1st, 2nd, 3rd, and 4th quartiles.

Injured athletes tended to experience more head impacts over the time delta during competition (median = 16 impacts, IQR 16.25 impacts) than preseason practices (median = 8 impacts, IQR 9 impacts) and regular season practices (median = 8 impacts, IQR 7.3 impacts) (Fig. 4), although those differences were not statistically significant. When comparing the differences in RWE between the three session types, athletes tended to accumulate more RWE during competitions (median = 0.024, IQR 0.1) compared to preseason practices (median = 0.011, IQR 0.02) and regular season practices (median = 0.009, IQR 0.02) (Table 2). While not significant differences, these may indicate that during competitions, athletes sustain more head impacts than the other session types, and those head impacts are of a higher magnitude. Although not significant, at the time of concussion, head impact accrual rate over the time delta in pre-season practices (median = 0.16 impacts/min, IQR 0.2 impacts/min) and regular season practices (median = 0.25 impacts/min, IQR 0.15 impacts/min) were both somewhat higher that the median rate during competitions (0.14 impacts/min, IQR 0.1 impacts/min).

**Table 2:** Median (IQR) and *p* values from the inter athlete analysis on session type.

Output variables	Session type			*p* values
	Competition	Preseason practice	Regular season practice	
	(*n* = 16)	(*n* = 29)	(*n* = 16)	
Linear acceleration magnitude (g)	62.8 (52.1)	55.1 (37.7)	54.2 (47.3)	0.58
Rotational acceleration magnitude (rad/s^2^)	3068 (2873)	2650 (1361)	2172 (1881)	0.45
Time delta length (min)	113.65 (99.1)	58 (60.4)	39.84 (40.9)	0.002
TD head impacts	16 (16.25)	8 (9)	8 (7.3)	0.052
TD head impact frequency percentile	79 (35)	72 (51)	72 (37.5)	0.94
TD head impact rate at injury (impacts/minute)	0.14 (0.1)	0.16 (0.2)	0.25 (0.15)	0.069
TD head impact rate percentile	49 (37)	39 (40)	50 (32.5)	0.85
TD RWE	0.024 (0.11)	0.011 (0.02)	0.009 (0.02)	0.16
TD RWE percentile	86 (31.8)	85 (37)	66 (50.3)	0.21
TD RWE rate	0.0002 (0.001)	0.0002 (0.001)	0.0002 (0.002)	0.95
(RWE/min)				
TD RWE rate percentile	75 (32.8)	71 (66)	71 (36.5)	0.98

Linear and rotational acceleration magnitudes are for the concussive head impacts

*TD* time delta; *RWE* risk-weighted exposure; percentile refers to the percentile of exposure on the concussion date relative to all the contact sessions for the injured athlete during the season of injury

## Discussion

The effects of repetitive subconcussive HIE sustained during contact sports have gained significant attention in recent years. Originally proposed as a modulator for reducing incident concussion tolerance, more recent studies implicated high levels of subconcussive HIE as a separate and independent mechanism of concussion.^56, 59^ For example, a recent study from the NCAA-DOD CARE Consortium reported that 72% of concussed athletes had evidence of elevated subconcussive HIE leading up to their concussion, with greater than half of those having no evidence of a high magnitude concussive impact.^56^ Variations in season-long HIE are thought to contribute, at least in part, to individual-specific concussion thresholds.^50^ Another recent study identified a prolonged effect of preseason HIE on season-long concussion incidence.^54^ Effects of HIE may also last beyond the time of contact sport participation, with high levels of cumulative HIE hypothesized to contribute to life-long cognitive and emotional issues.^1, 42^ Beyond its role in incident concussion, accumulating clinical changes associated with high levels of HIE may also occur in athletes that do not report concussive injury.^3^ In fact, significant cognitive dysfunction was identified in contact sport athletes that sustained high levels of HIE through routine participation in contact sports without diagnosed concussion.^39, 42, 58^ Recent preclinical modeling also identified significant dose-dependent behavioral changes in rats receiving repeated low-level head accelerations scaled from human contact sport HIE^52^ or repeated ‘mild’ exposures.^8, 47, 52, 55^ From a mechanistic standpoint, imaging studies identified changes in brain white matter integrity that were positively correlated to levels of HIE.^4, 20, 21^ Preclinical studies highlighted neuroinflammatory responses,^52^ as well as dose-dependent microgliosis and axonal damage that were correlated to the level of HIE.^55^ Chronic studies identified higher later-life t-tau concentrations in former professional football athletes that had sustained higher estimated levels of HIE.^2^ These studies have formed the foundation of our understanding of how repetitive subconcussive HIE influences concussion and concussion-like symptoms. The present study adds to this ever-expanding body of knowledge.

However, aside from preclinical studies that provided definitive evidence of pathophysiology and behavioral changes linked to repetitive HIE,^55^ some debate remains regarding the mechanistic role of subconcussive HIE in acute sport-related concussion or the development of concussion-like symptoms in uninjured athletes. In 2016, Belanger *et al*. published a review on the contemporary evidence linking HIE to acute clinical outcomes.^7^ That study concluded that subconcussive blows were not shown to cause significant clinical effects and that any effect of subconcussive blows is likely to be small or nonexistent. Other studies supported that finding by demonstrating a lack of strong correlation of concussion biomechanics and HIE with acute clinical outcomes.^41, 50^ Mihalik *et al*. indicated that season HIE or injury day HIE did not predict clinical recovery trajectories for symptom severity, balance, or mental status, despite greater HIE being associated with increased symptom resolution time and shorter return to play times.^41^ However, factors such as playing position, offensive/defensive scheme, personal concussion/HIE history, and genetics may influence concussion tolerance. Therefore, to eliminate those factors, the present analysis was focused on comparisons of injury date HIE to an athlete’s own HIE throughout the season.

In the present analysis, we predicted that HIE would be elevated in athletes on the concussion date relative to their HIE in other contact sessions for that season. Present findings supported this hypothesis and demonstrated that a high percentage of athletes experienced elevated HIE over the time delta than over the same duration for other contact sessions during their season of injury. For example, approximately 29.5% of concussed athletes sustained a head impact frequency on the injury date that ranked in the 90th percentile or higher when compared to the distribution of head impact frequencies sustained over the time delta in all other contact practices and games during that season. For 11% of athletes, the number of head impacts sustained over the time delta was the highest of all sessions during their season of injury. These important findings provide more evidence for the role of HIE in concussion biomechanics for contact sport athletes and highlight the need to analyze concussion and HIE data on an individual basis.

These findings lend insight into the mechanism by which HIE contributes to incident concussion in contact sports. The present focus on the number of head impacts and RWE over a fixed time approximates HIE frequency. Accordingly, a greater number of head impacts over the fixed time delta on the concussion date provides preliminary evidence that increased HIE frequency may influence concussion onset. For example, the median time delta head impact rank was in the 73rd percentile, indicating that on the day of injury, most athletes had elevated HIE over the time delta compared to any other time delta period throughout the season of injury. This was also the case for RWE accumulation over the time delta as the median rank was in the 85th percentile. If HIE frequency over the fixed time delta had no effect, it would be expected that the dataset be normally distributed, meaning only 16 athletes (27%) would be expected to have their time delta impacts rank at or above the 73rd percentile, however, 31 (50.8%) athletes were at or above this ranking. The same phenomenon is seen in the total RWE analysis, where 31 athletes were at or above the median rank of the 85th percentile compared to the 9 athletes (15%) that would have been expected to be at or above that ranking in a normally distributed population.

While the time to concussion was significantly different between the three session types and HIE metrics were non-significantly different between session types (Table 2), the main outcomes of this analysis [time delta (TD) percentile metrics] were not different between session types including head impact frequency percentile (*p* = 0.94), head impact rate percentile (*p* = 0.85), RWE percentile (*p* = 0.16), and RWE rate percentile (*p* = 0.98).

Similar to the present analysis, Broglio *et al*. also found elevated exposure on the day of injury prior to concussion, reporting that the population of concussed athletes in their analysis had shorter time intervals between head impacts on the injury date, and those impacts were higher in magnitude compared to matched controls.^10^ Beckwith *et al*. reported a higher number of head impacts on the concussion date in a concussed population,^6^ but unlike that study, the present HIE frequency analysis was conducted on an intra-athlete basis, demonstrating that athletes had elevated HIE frequency on the concussion date based on the time delta metric. Previous findings by Eckner *et al*. conflict with the present results, as no difference was found in the frequency prior to concussion compared to HIE before other high magnitude head impacts. However, this may be attributed to the methods they employed, which involved counting impacts only in the 30 min prior to concussion, and finding no difference as compared to past exposure history.^24^ The present study did not have a time cutoff for counting exposure on the injury date, and athletes time delta lasted for a median of 58.8 min after their first head impact, nearly double the cutoff time of Eckner and associates. This suggests head impacts could have been omitted from their injury date analysis that may have changed their findings to be more in line with the present findings.

Elevated exposure levels seen in concussed athletes compared to other time delta periods throughout the season in which they do not sustain an injury suggests that, for an individual athlete, concussion tolerance may be reduced following periods of elevated HIE frequency. These findings are similar to the results of Rowson *et al*., who found that the concussive head impact was one of the hardest impacts an athlete experienced during the season of their injury (Fig. 3).^50^ As Rowson first suggested, and as was found from the present analysis, a case can be made for future HIE and concussion studies to primarily focus on the data for each individual and not the entire athlete population. This is a logical next step as other studies identified significant variation in HIE between individuals.^57^ It was shown that concussive impact mechanics are unique for individuals and the same can be said for the level of exposure experienced by an athlete sustaining concussion. In fact, variance in concussive impact mechanics may be due to a combined effect wherein pre-concussion HIE reduces the tolerance for concussion associated with individual impacts. According to this theory, greater pre-concussion HIE would lead to a pronounced decrease in tolerance.

In addition to acute mechanical aspects, concussion history may influence the risk of incident concussion.^27^ This was briefly analyzed within the present population to determine if concussion history affected exposure levels at concussion. No differences were found for any of the analyzed metrics between athletes were grouped by those with (*n* = 26) and without (*n* = 35) concussion history. This is not surprising as prior studies reported similar findings.^50^ Other factors, including biological and psychological aspects such as genetics and resilience may help to explain intra-athlete variance in concussive impact magnitudes.^27, 31, 35, 43, 51^ With these factors known, a unique risk profile could be developed for each athlete which would account for an individual’s exposure, concussion history, past head impact magnitudes, and biological risk factors, allowing athletic medicine personnel to better identify athletes at risk of concussion prior to participation using concurrent data from helmet-based or head-mounted sensors and to assist in concussion diagnosis.

The present population offers insight into the number of athletes that could benefit from such a diagnostic risk curve as 63% (37 concussions) of the athlete population sustained ongoing head impacts for an extended period of time after concussive injury (> 5 min). In addition to continued HIE accumulation, delayed removal could have introduced error into the dataset when determining the injurious head impact. To quantify the effect of this on HIE metrics, the entire analysis (originally performed with 61 concussions) was repeated for two limited subgroups wherein (1) athletes were removed within 5 min of the concussive head impact (22 concussions), and (2) athletes were removed immediately after the concussive impact (12 concussions) (Table 3). Athletes who remained in play had lower HIE metrics than those who were rapidly removed from play. Those that were removed within 5 min showed similar results to the entire population, and the analysis of those with no further head impacts showed an even stronger indication of the influence of TD exposure.

**Table 3:** Comparison of all athletes regardless of when they were removed from play after injury to athletes that were removed after less than 5 min of play and athletes that were removed immediately after the injurious head impact.

	All athletes	Athletes removed less than 5 min	Athletes removed immediately
Number of athletes	61	24	17
Median TD head impacts percentile	73	84	86
Median TD head impact accrual rate percentile	48	49.5	50
Median TD RWE percentile	85	89.5	95
Median TD RWE accrual rate percentile	74	61.5	71

Our choice of head impact measurement systems was based on two important factors: the ability of the system to accurately record individual head impacts in football athletes and the ability of the system to support large-scale deployment and data collection at remote sites. At the time of our study initiation, the HIT System represented the best available technology for the scale of data collection required by the CARE Consortium HIM. The HIT System has been in use for more than 15 years and its ability to accurately measure sport-related head impacts was evaluated several times since its introduction.^5, 19, 23, 33, 53^ Those evaluations reported 78 to 98% positive head impact identification rates,^5, 23, 33, 53^ + 0.9 to + 29.5% relative error for linear acceleration magnitudes,^5, 19, 23, 33, 53^ and − 23 to + 76% relative error for rotational acceleration magnitudes.^5, 19, 23, 33, 53^ Reduced rotational acceleration accuracies may be attributed, at least in part, to the HIT System only incorporating five degrees of freedom. The system algorithm necessarily constrains *z*-axis rotational acceleration (i.e., rotational acceleration about the anatomical superior–inferior axis) to zero. Therefore, the HIT System may have reduced accuracy for head impacts producing appreciable *z*-axis rotational accelerations. An example would be a horizontal impact to the side of the facemask causing the head to rotate laterally to the athlete’s right or left. Nonetheless, experimental data indicate that the HIT System accurately detected individual head impacts despite lacking a degree of freedom, although the magnitudes of those impacts were reported in some studies to be less accurate. In additional to *z*-axis rotational acceleration discussed above, variability in the accuracy of head impact magnitudes between studies may also be due, in part, to helmet model used, a lack of consistency in helmet size/fit, and differing methods used to achieve proper helmet fit.

The 50th percentile Hybrid III (H3) headform, used in each of the validation studies, was initially developed for automobile testing,^32^ and it’s anatomical features do not lend themselves to proper helmet fit.^18^ Those features may lead to excessive helmet motion and poor force dissipation during impact, leading to head acceleration overestimation identified in some validation studies. For example, because the H3 headform surface does not model the head to neck transition, the posterior aspect of the headform does not stay in contact with the bottom portion of helmet padding;^18^ the H3 headform has much thinner cheek bones, resulting in less contact with the cheek pads compared a football player wearing a properly fitted helemet;^18^ the chin does not protrude as far out as a chin found on a 50th male finite element model,^26^ which results in a smaller moment arm to arrest helmet motion via the chin strap during a frontal impact compared to the more biofidelic male model.^18^ One validation study used a medium helmet on the H3 headform, which has a tighter fit on the headform compared to how humans wear their helmets and are too small for the circumference of the H3 headform per the manufacturers fit instructions.^5, 33^ However, this tighter fit may be more representative of proper human fit due to the anatomical issues of the H3 headform. Siegmund and colleagues attempted to account for some of the differences in the cheek bone and chin geometry by using a modified H3,^53, 62^ but did not account for occipital geometrical differences which may have contributed to acceleration magnitude errors.

Present findings should be interpreted in light of these laboratory-based outcomes. Similar to our previous studies,^56^ head impact counts and RWE had similar outcomes. Current results indicated that approximately 21% of concussed athletes had a frequency of head impacts on their concussion date that was above the 95th percentile for any similar time period of HIE for that athlete during their season of injury. That percentage was somewhat higher (31 vs 21%) for RWE. Approximately 48 and 57% of concussed athletes were above their 75th percentile for impact count and RWE on their concussion date. While RWE findings were stronger in the current analysis, two important points should be made. Firstly, the similarity between impact count and RWE indicates that impact count is likely a primary underlying factor in altering concussion tolerance for contact sport athletes. Secondly, laboratory-based variability in peak acceleration measurements was partly a function of helmet fit. Improper helmet fit can lead to less rigid coupling to the head and increased error in acceleration measurements. The current analysis compared each concussed athlete’s HIE on their concussion date to their own HIE throughout the season, thus removing variability associated with different helmet fits between athletes. Additionally, at the NCAA Division I level, there would be an assumption that each athlete would have an appropriately sized and fitted helmet. Therefore, these results are presented with increased confidence in the accuracy of our findings as presented and their interpretation for the role of HIE in the concussion mechanism.

Recent studies and research protocols called for video verification of head impacts to confirm the on-field accuracy of head impact sensors.^16, 34, 36^ To date, one study completed such an analysis for the HIT System by analyzing special teams plays from high school football competitions.^14^ That study reported an 88% positive predictive value, meaning the HIT System underestimated the total number of head impacts seen on video by 12%, similar to laboratory-based findings.^5, 14, 23, 33, 53^ We assume the positive predictive value to be accurate for our analysis as well but are not able to quantify the effect of including more than just special teams plays (i.e., practices), which often include more varied activities. A separate video verification reported that the HIT System outperformed all other tested sensors in head impact detection rate, with the exception of a custom-made mouthguard system.^34^ While video verification is useful in determining metrics such as the positive predictive value, it lacks the ability to verify acceleration magnitudes. Laboratory-based errors in the HIT System acceleration magnitudes should be interpreted with caution given the variety of factors that can influence accuracy of that system including helmet fit, which can vary between human and surrogate headforms. Based on the information presented, and its use in literature, some level of inaccuracy is present in the HIT System dataset and results dependent on acceleration magnitudes should be interpreted with that knowledge. In this analysis and our ongoing research, we attempted to reduce the influence of those inaccuracies by comparing athlete biomechanics on their concussion date to their own non-injury biomechanics to reduce the effect of helmet fit variability and focusing to a higher degree on impact counts with less of a focus on the magnitudes of individual head impacts.

Video verification is an important part of head impact data collection using head-mounted sensors. While we attempted to video verify head impacts in this study, we were limited by a number of factors and eventually were not able to do so for non-concussive events. For example, coaching staffs were not willing to share their practice videos for competitive reasons and television game broadcasts provide significant barriers to game video verification with regard to time syncing, field of view, and players that move in and out of frame. These are issues that complicate on-field video verification across the vast majority of studies that are performed in large samples (i.e., more than one team) and at higher levels of competition (collegiate and above). Video verification is extremely useful where it can be properly incorporated. However, we must acknowledge that existing on-field video verification studies for football were conducted in controlled and limited environments^14, 34, 36^ (i.e., special teams, small enrollment, *etc*.). Given that the same system is used across multiple studies, it may be most appropriate to use these complementary goals (small scale video verification in some studies with large scale data collection in others) to understand system accuracy/limitations while providing large-scale data that is needed to characterize generalizable injury biomechanics.

This analysis was limited by several factors. First, some non-injury sessions that were shorter in total duration than the athlete’s concussion day time delta were included, as our objective was to compare concussion day exposure to all non-concussion days. However, inclusion of shorter days may artificially increase relative exposure on the concussion day. If those sessions were removed, removing 34.8% (882 sessions) of non-concussed days from the analysis, similar trends were present to the data presented above (Table 4). This secondary analysis clearly demonstrates the robustness of our findings in that the vast majority of concussed athletes had a greater number of head impacts and greater RWE on their concussion date compared to the other contact days for their season of injury in which they did not sustain concussion. That was true whether we compared the concussion date to all non-injury days or only non-injury days that had a duration at least as long as the time delta.

**Table 4:** Comparison of all non-injury sessions to only non-injury sessions that were equal to or longer in duration than an athlete’s time delta (TD).

	All non-injury sessions	Only non-injury sessions longer than TD
Number of athletes with non-concussed sessions	61	60
Non-concussed days	2537	1655
Median TD head impacts percentile	73	70
Athletes above the 75th percentile	29	27
90th percentile	18	17
95th percentile	13	14
99th percentile	7	8
Median TD head impact accrual rate percentile	48	48
Athletes above the 75th percentile	8	9
90th percentile	2	5
95th percentile	1	3
99th percentile	0	1
Median TD RWE percentile	85	73
Athletes above the 75th percentile	35	30
90th percentile	25	22
95th percentile	19	15
99th percentile	11	11
Median TD RWE accrual rate percentile	74	61
Athletes above the 75th percentile	28	22
90th percentile	15	14
95th percentile	12	9
99th percentile	6	7

Second, despite being one of the largest national studies on sport-related concussions, the total number of concussions was still somewhat limited. As with all analyses of this type, a greater number of concussions would allow for a more in-depth statistical analysis. Despite this limitation, the trends found within the analysis, indicate that the dataset was significant enough to make the conclusions presented.

Unlike prior analyses of HIE frequency which looked at the entire concussion date or included other days prior to the concussion,^6, 10, 24^ the present analysis was limited to analyzing the HIE an athlete sustained on their day of injury from the first head impact to the concussive head impact. Given the proposed causative relationship of HIE on concussion tolerance, we felt it more important to analyze HIE prior to concussion than exposure levels across the entire injury date.

Finally, as previously mentioned, the HIT System is only a five degree of freedom system and has other limitations previously mentioned. While it was the best option when our study was initiated, HIM technology has advanced significantly, and commercial devices now exist with a sixth degree of freedom,^34, 37^ better coupling to the skull for more accurate head impact kinematics,^63^ and deployable for a large scale study. These improved devices should be utilized in future head impact measurement studies moving forward.

This analysis showed that concussed athletes had both elevated HIE frequency and risk weighted exposure on their concussion day. This indicates that on the day of injury, prior to a concussion, an athlete experienced an elevated number of head impacts and the combined risk of those impacts were elevated compared to days in which an athlete did not sustain a concussion. The findings of this analysis support the hypothesis of HIE as a second mechanism of concussion and provides evidence that an increase in HIE frequency may influence concussion onset. These findings can be used to develop a protocol to monitor in-session exposure levels to potentially identify athletes that may be susceptible to incident concussion.
